# Alternative to Poly(2-isopropyl-2-oxazoline) with a Reduced Ability to Crystallize and Physiological LCST

**DOI:** 10.3390/ijms22042221

**Published:** 2021-02-23

**Authors:** Wojciech Wałach, Agnieszka Klama-Baryła, Anna Sitkowska, Agnieszka Kowalczuk, Natalia Oleszko-Torbus

**Affiliations:** 1 Centre of Polymer and Carbon Materials, Polish Academy of Sciences, 34 M. Curie-Skłodowskiej St., 41-819 Zabrze, Poland; wwalach@cmpw-pan.edu.pl (W.W.); akowalczuk@cmpw-pan.edu.pl (A.K.); 2 Dr. Stanislaw Sakiel Center for Burn Treatment, 2 Jana Pawla II St., 41-100 Siemianowice Slaskie, Poland; agawa777.78@gmail.com (A.K.-B.); sitkowska-anna@wp.pl (A.S.)

**Keywords:** poly(2-isopropyl-2-oxazoline), crystallization, thermal properties, LCST

## Abstract

In this work, we sought to examine whether the presence of alkyl substituents randomly distributed within the main chain of a 2-isopropyl-2-oxazoline-based copolymer will decrease its ability to crystallize when compared to its homopolymer. At the same time, we aimed to ensure an appropriate hydrophilic/lipophilic balance in the copolymer and maintain the phase transition in the vicinity of the human body temperature. For this reason, copolymers of 2-ethyl-4-methyl-2-oxazoline and 2-isopropyl-2-oxazoline were synthesized. The thermoresponsive behavior of the copolymers in water, the influence of salt on the cloud point, the presence of hysteresis of the phase transition and the crystallization ability in a water solution under long-term heating conditions were studied by turbidimetry. The ability of the copolymers to crystallize in the solid state, and their thermal properties, were analyzed by differential scanning calorimetry and X-ray diffractometry. A cytotoxicity assay was used to estimate the viability of human fibroblasts in the presence of the obtained polymers. The results allowed us to demonstrate a nontoxic alternative to poly(2-isopropyl-2-oxazoline) (PiPrOx) with a physiological phase transition temperature (LCST) and a greatly reduced tendency to crystallize. The synthesis of 2-oxazoline polymers with such well-defined properties is important for future biomedical applications.

## 1. Introduction

**Figure 1:**
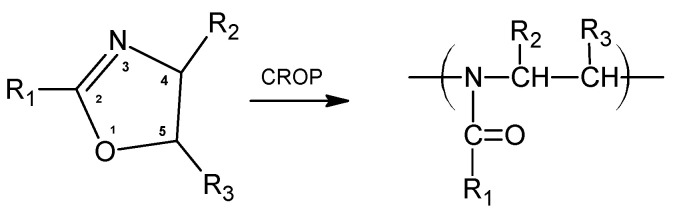
The 2-Oxazoline polymerization scheme.

Stimuli-responsive polymers showing a reversible response to external stimuli have attracted much attention as “smart” and advanced materials in the last few decades [1]. In particular, thermoresponsive polymers exhibiting a phase transition around body temperature are of special interest due to their potential for biomedical applications. Among thermoresponsive polymers with a peptidomimetic structure, exhibiting a lower critical solution temperature (LCST) at the physiological level, poly(N-isopropylacrylamide) (PNIPAM) and poly(2-isopropyl-2-oxazoline) (PiPrOx) are widely known. PNIPAM is considered the “gold standard” of LCST polymers for biomedical applications [2]. The interesting property of this polymer is the low sensitivity of its LCST to environmental conditions (variations in pH, concentration or the chemical environment affect the LCST of PNIPAM by only a few degrees [3,4]). When heated, the PNIPAM aqueous solution exhibits a sharp phase transition; however, an undesired broad hysteresis can be observed during cooling, which is involved with the irreversible coil-to-globule transition [5]. PiPrOx is the structural isomer of PNIPAM, and it exhibits no hysteresis of the phase transition [6,7]. PiPrOx is a member of a large group of polymers known as poly(2-oxazoline)s that are obtained via cationic ring opening polymerization (CROP) of five-membered cyclic imino ethers containing a double bond at position 2 (Figure 1) [8].

The nature of substituents R_1_, R_2_ and R_3_ determines the poly(2-oxazoline) properties. PiPrOx with the isopropyl substituent R_1_ is known to be a thermoresponsive polymer [9] exhibiting nontoxicity toward a variety of cell lines [10,11,12,13] and thus is discussed as an alternative to PNIPAM. The dependence of the phase transition temperature on the molar mass or the presence of a surfactant was found to be more pronounced for PiPrOx than for PNIPAM [7,14]. Another unique feature of PiPrOx is its tendency to crystallize upon the prolonged incubation at an elevated temperature and the formation of hierarchically structured objects [15,16,17]. However, this property seems detrimental for certain, mainly biomedical, applications. Hence, the goal of the present study was to obtain an alternative to PiPrOx with a reduced ability to crystallize. It was important to carry out the modification so that the obtained polymer was nontoxic and exhibited a LCST close to the human body temperature.

The chemical structure of PiPrOx (with the isopropyl substituent R_1_ attached to the planar amide groups) favors easy packing and, as a consequence, ordering of the polymer chains. Crystallization of homo- and copolymers of 2-isopropyl-2-oxazoline (iPrOx) in the solid-state [18], in aqueous solutions [15,17,19,20,21,22,23,24,25,26] and in organic [27] and water/organic mixtures [16] has been described in great detail. Efforts were made to reduce the ability of PiPrOx to crystallize. For that purpose, the introduction of additional 2-oxazoline comonomers with R_1_ substituents other than isopropyl was carried out to disrupt the regularity of the PiPrOx chain. Recently, it was reported that the copolymerization of iPrOx with 2-methyl-2-oxazoline (MetOx) caused a decrease in the ability of the copolymer to crystallize when compared to PiPrOx. The LCST of the obtained copolymers changed significantly, and moreover, prolonged incubation of the iPrOx/MetOx copolymers at an elevated temperature caused further crystallization [28]. The other approach utilized the copolymerization of iPrOx with its structural isomer 2-n-propyl-2-oxazoline (nPrOx), which led to a decrease in the ability of the copolymer to crystallize and a slight decrease in LCST (only of a few °C) when compared to PiPrOx. Unfortunately, the prolonged heating of iPrOx/nPrOx also caused further crystallization, similar to the case of the iPrOx/MetOx copolymers [25]. Attempts to introduce ethylene imine (EI) units within the iPrOx-copolymer chain led to the suppression of crystallization even upon prolonged annealing, which was attributed to the increased elasticity of the chains induced by EI segments, but simultaneously, a significant change in the LCST was observed [28].

Based on these studies, it seems that by controlling only the R_1_ substituent of the comonomer, it is not possible to disorder the chain regularity of iPrOx-based copolymers and, consequently, to suppress its ability to crystallize, with simultaneous control of the LCST at the desired level.

In this work, we aimed to synthesize copolymers of iPrOx with 2,4-disubstituted-2-oxazoline and to check whether, by an appropriate selection of R_1_ and R_2_ substituents of the comonomer, it is possible to suppress the crystallization of the copolymer while simultaneously maintaining the LCST close to the human body temperature. For this reason, we carried out the copolymerization of iPrOx with 2-ethyl-4-methyl-2-oxazoline (EtMetOx). We expected that the methyl substituent R_2_ present within the main chain of the iPrOx copolymer would disorder the chain-specific arrangement, which is responsible for the ability of the copolymer to crystallize. At the same time, we expected that the ethyl substituent R_1_ would ensure an appropriate hydrophilic/lipophilic balance and an LCST near the physiological temperature. The cytotoxicity of the copolymer must be tested to check its bioapplicability. The synthesized polymers were found to be nontoxic to fibroblasts at the concentrations required for biomedical tests. Such noncrystalline copolymers of 2-oxazolines exhibiting a phase transition around body temperature have not been obtained previously and could be interesting candidates for the design of polymeric nanosystems for a wide range of biomedical applications.

## 2. Results and Discussion

As claimed, we aimed to introduce an alkyl substituent into the main chain of the iPrOx copolymer to disorder its regularity and specific arrangement, which should subsequently decrease its ability to crystallize. For this reason, copolymerization of iPrOx with 2,4- or 2,5-disubstituted-2-oxazoline is necessary. CROP of 2-oxazolines substituted with R_2_ leads to a higher conversion of the monomer when compared to 2-oxazolines with R_3_. Usually, 2-oxazolines with R_2_ substituted by methyl [29,30,31] or ethyl [17,32,33,34,35] groups are used, but the polymerization of 2-oxazolines with –C(O)OCH_3_ or phenyl [35] R_2_ substituents is also known. As, at the same time, we aimed to assure the LCST at a temperature close to the physiological value, the role of the R_1_ substituent of the comonomer is also crucial. To provide an appropriate hydrophilic/lipophilic balance of the copolymer, the proper length of the R_1_ alkyl substituent must be selected and the amount of the comonomer must be controlled.

**Figure 2:**
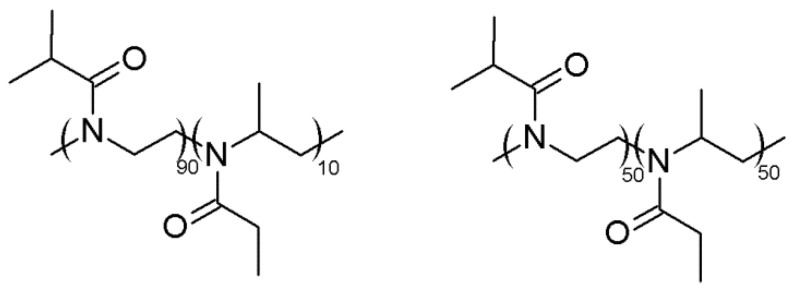
Structure of the copolymers of 2-ethyl-4-methyl-2-oxazoline (EtMetOx) and 2-isopropyl-2-oxazoline (iPrOx).

For this reason, copolymers of 2-ethyl-4-methyl-2-oxazoline (EtMetOx) and iPrOx with different contents of EtMetOx were synthesized via CROP. The theoretical degree of polymerization (DP) was assumed to be 100 and the copolymer composition was assumed with a DP of EtMetOx of 10 or 50. The chemical structure of the copolymers is presented in Figure 2.

Based on the monomer consumption rate, copolymers of the random structure were obtained. The DP of the macromolecules was calculated on the basis of the conversion of comonomers and NMR data, and the copolymers were denoted as P(EtMetOx_10_-iPrOx_90_) and P(EtMetOx_50_-iPrOx_50_). Good agreement of the molar mass with the theoretical value was achieved, as obtained from GPC-MALLS (M_n_ = 13,000 g mol^−1^, *Ð* = 1.14 and M_n_ = 8500 g mol^−1^, *Ð* = 1.34, respectively) (Supplementary Figures S1 and S2). A panel of properties (crystallization in aqueous solution and in the solid-state) was analyzed for the first copolymer P(EtMetOx_10_-iPrOx_90_) due to its transition temperature being close to the human body temperature (see below). The second copolymer P(EtMetOx_50_-iPrOx_50_) was used for cytotoxicity studies as its transition temperature ensured the solubility of the polymer in the medium at culture conditions.

### 2.1. Properties in the Aqueous Solution

**Figure 3:**
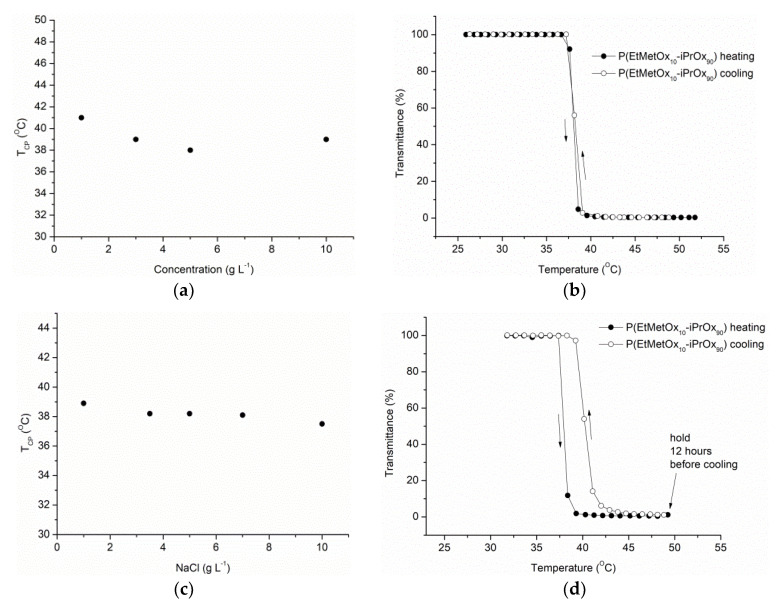
(**a**) Plot of T_CP_ as a function of the P(EtMetOx_10_-iPrOx_90_) concentration, (**b**) transmittance-temperature dependence of the aqueous solution of the copolymer (c = 5 g L^−1^), (**c**) plot of T_CP_ as a function of the NaCl concentration (the copolymer concentration in water is 5 g L^−1^) and (**d**) transmittance-temperature dependence of the aqueous solution of the copolymer (c = 5 g L^−1^) kept for 12 h at 50 °C before cooling.

Thermosensitivity studies by turbidimetry revealed that the LCST of P(EtMetOx_10_-iPrOx_90_) was equal to 38 °C, which is the same as that of PiPrOx [14] (Figure 3a). The phase transition of the copolymer was completely reversible. When the polymer solution was heated above the cloud point temperature (T_CP_), given as 50% transmittance at the heating cycle, and then cooled to room temperature, practically no hysteresis of transition was observed (Figure 3b). An important factor influencing the biomedical applicability of polymers is the presence of various salts in the solution and their effect on the thermosensitivity of the polymers [36,37]. Figure 3c shows the cloud points of the copolymer recorded in the presence of increasing amounts of sodium chloride. A slight salting-out effect is observed as the presence of sodium chloride leads to a natural partial dehydration of the polymer; however, the change in T_CP_ is rather subtle, similar to the case of PiPrOx [38]. In conclusion, when short-term heating conditions are applied, P(EtMetOx_10_-iPrOx_90_) exhibits a thermoresponsive behavior comparable to that of PiPrOx.

Incubation of P(EtMetOx_10_-iPrOx_90_) in water at a temperature above T_CP_ (50 °C) for 12 h led to a full return to transparency of the solutions when the temperature was decreased (Figure 3d). Macroscopically, this fact indicates a lack of crystallization in the solution, in contrast to PiPrOx, where the solution after incubation under similar conditions and cooling remained cloudy and the separated precipitate was crystalline [15,39]. However, a hysteresis of the phase transition of approximately 2 °C was found for the copolymer incubated for a prolonged time at temperature above T_CP_.

Briefly, P(EtMetOx_10_-iPrOx_90_), similar to PiPrOx, exhibits a LCST near the physiological temperature and it is slightly dependent on the concentration and salts. Importantly, the copolymer, in contrast to PiPrOx, does not crystallize in water when long-term heating conditions are applied.

### 2.2. Properties in the Solid-State

The thermal and crystalline properties of P(EtMetOx_10_-iPrOx_90_) in the solid-state were studied by DSC and WAXS.

**Figure 4:**
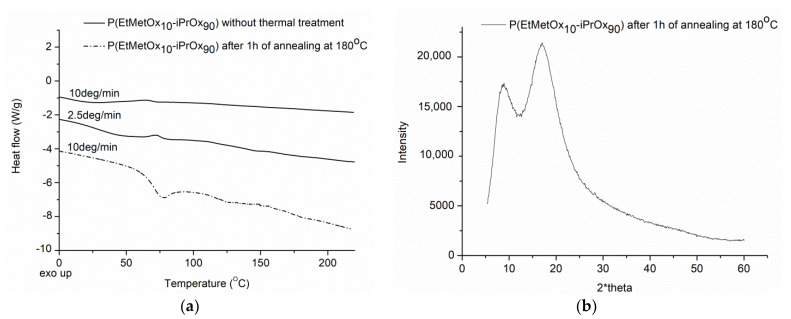
(**a**) DSC traces and (**b**) X-ray diffraction curve of P(EtMetOx_10_-iPrOx_90_).

It can be seen that neither crystallization nor melting is observed during the standard (10 °C/min) or even slow (2.5 °C/min) DSC measurements, indicating the low tendency of this copolymer to crystallize (Figure 4a). Moreover, after thermal treatment (annealing of the copolymer at 180 °C for 1 h, and then cooling to room temperature at a rate of 10 °C/min), the lack of exo- or endothermic peaks can be seen in the DSC trace. Such behavior is different from that of PiPrOx, where the substantial effects from crystallization at ~150 °C and melting at ~200 °C were observed in the DSC traces [16,27].

The glass transition temperature (T_g_) of the copolymer, determined from measurement after the first run and quenching with liquid nitrogen, was equal to 68 °C, which is the same as that for PiPrOx [27,39] (Supplementary Figure S3). Additionally, no diffraction peaks can be seen in the WAXS curve for P(EtMetOx_10_-iPrOx_90_) (Figure 4b), confirming the conclusion drawn from the DSC that the amorphous phase is predominant, even after 1 h of annealing of the copolymer. In contrast, two characteristic diffraction peaks at 2θ = 7.84° and 18.08° could be seen in the WAXS curve for PiPrOx [39]. Additionally, we checked whether the very prolonged annealing of the copolymer (for 12 h) at 180 °C and further slow cooling would enhance its ability to crystallize. While no diffraction peaks could be seen in the WAXS curve (Supplementary Figure S4a), small exo- and endothermic peaks appeared in the DSC trace, with maxima at 155 °C and 188 °C, respectively (Supplementary Figure S4b). These effects exhibit very low energy (ΔH ~0.5 J/g); nevertheless, such behavior indicates that it is not possible to completely neglect the ability of seemingly amorphous polymers to crystallize. To conclude, in the solid-state, P(EtMetOx_10_-iPrOx_90_) exhibits a definitely reduced ability to crystallize when compared to PiPrOx, whilst maintaining a T_g_ similar to that of PiPrOx.

### 2.3. The Role of the R_2_ Methyl Substituent

We assume that the reduced ability of P(EtMetOx_10_-iPrOx_90_) to crystallize compared to PiPrOx results from the presence of the methyl R_2_ substituent distributed within the main chain, while maintaining the LCST of the copolymer near physiological temperature results from the proper choice of an R_1_ (ethyl) substituent of the comonomer and the control of the amount of 2,4-disubstituted-2-oxazoline. The appropriate selection of R_1_ and R_2_ influences the thermosensitivity and ability to crystallize, clearly reflecting the structure–property relationships of poly(2-oxazoline)s.

**Figure 5:**
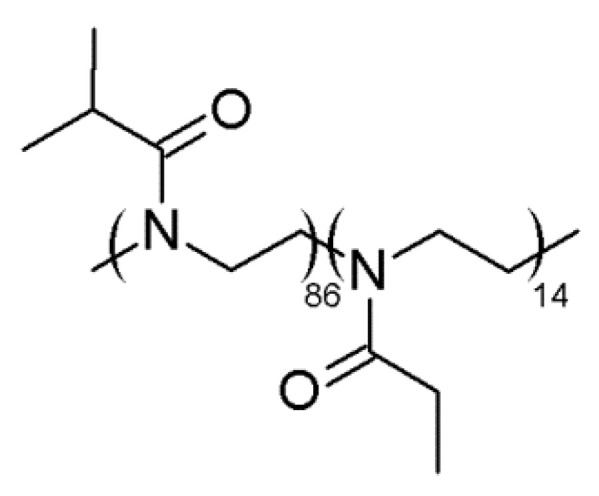
Structure of the copolymer of 2-ethyl-2-oxazoline (EtOx) and iPrOx.

To verify the key role of the R_2_ substituent in decreasing the ability of P(EtMetOx_10_-iPrOx_90_) to crystallize, we obtained the copolymer of iPrOx with 2-ethyl-2-oxazoline (EtOx) of a similar composition, and we compared the thermal and crystalline properties of copolymers with and without R_2_ methyl substituents within the main chain. The chemical structure of the copolymer is presented in Figure 5.

The characterization of P(EtOx_14_-iPrOx_86_) by ^1^H NMR, SEC-MALLS and turbidimetry is provided in the Supplementary Figure S5.

**Figure 6:**
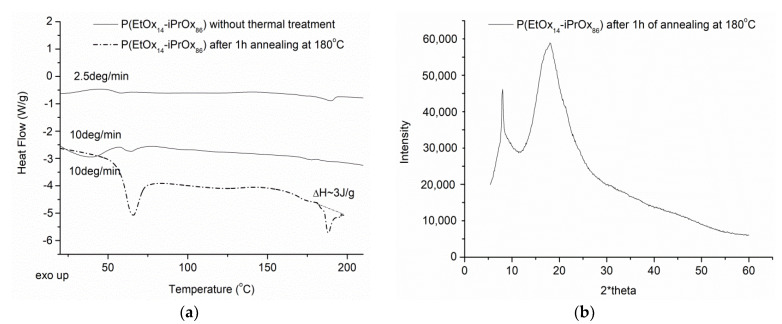
(**a**) DSC traces and (**b**) X-ray diffraction curve of P(EtOx_14_-iPrOx_86_).

In the DSC trace of P(EtOx_14_-iPrOx_86_) recorded at 2.5 °C/min, a small endothermic peak can be seen at ~185 °C, in which enthalpy significantly increased to ~3 J/g when the sample was thermally treated before measurement (annealing at 180 °C for 1 h and then cooling to room temperature at a rate of 10 °C/min) (Figure 6a). Additionally, the diffraction peak at 2θ ~8° can be seen in the WAXS curve of thermally treated P(EtOx_14_-iPrOx_86_) (Figure 6b).

Additionally, for P(EtOx_14_-iPrOx_86_) annealed for a much longer time (12 h) at 180 °C, a pronounced endothermic peak at ~185 °C (ΔH ~13 J/g) can be seen in the DSC trace (Supplementary Figure S6a), together with a significant diffraction peak at 2θ ~8° (Supplementary Figure S6b), indicating the considerable ability of this copolymer to crystallize. This finding confirms that the methyl R_2_ substituent strongly influences the disorder of the chain-specific arrangement of iPrOx-based copolymers, thus inhibiting crystallization.

### 2.4. Cytotoxicity

**Figure 7:**
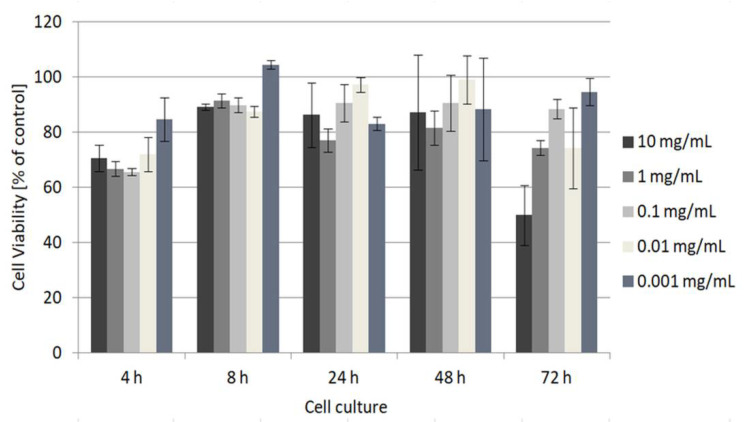
Cytotoxicity assay of P(EtMetOx_50_-iPrOx_50_) at increasing concentrations (given in mg/mL). The assay was performed with fibroblasts. The results are shown as a percentage of the control, where untreated cells constituted 100%.

The MTT test, a standard laboratory colorimetric assay for the measurement of cellular growth, was used to evaluate the cytotoxicity of the copolymer. P(EtMetOx_50_-iPrOx_50_) was used for the test due to its LCST of 41 °C, which means that during cell culture carried out at 37 °C, it is fully soluble in DMEM (Supplementary Figure S2c). In this study, human fibroblasts were selected as model cells. Human fibroblasts are commonly used to verify the cytotoxicity of polymers, and we have shown the lack of toxicity of (co)poly(2-oxazoline)s towards fibroblasts in our previous studies [12,13]. The concentrations of the copolymer were as follows: 0.001, 0.01, 0.1, 1 and 10 mg/mL. The results are shown in Figure 7.

P(EtMetOx_50_-iPrOx_50_) is nontoxic to fibroblasts in a wide range of concentrations. The percentage of fibroblast viability was at least 70%, relative to the control, except for the highest concentration (10 mg/mL) at 72 h. The obtained results indicate that synthesized polyoxazolines may be considered for future applications in biomedicine.

## 3. Materials and Methods

### 3.1. Materials

Isobutyronitrile (99.6%, Aldrich, Steinheim, Germany), 2-aminoethanol (99%, Aldrich, Steinheim, Germany), cadmium acetate (>98%, Fluka, Steinheim, Germany), D,L-Alaninol (98%, Aldrich, Steinheim, Germany), propionitrile (>99%, Fluka, Steinheim, Germany) and methyl 4-nitrobenzenesulfonate (99%, Aldrich, Steinheim, Germany) were used as received. 2-Ethyl-4-methyl-2-oxazoline (EtMetOx) was synthesized according to Schubert [17]. 2-Isopropyl-2-oxazoline (iPrOx) was synthesized according to Witte and Seeliger [40]. Raw EtMetOx, iPrOx and EtOx (99%, Aldrich, Steinheim, Germany) were dried over KOH, distilled, dried over CaH_2_ and distilled again. Acetonitrile (for HPLC, POCH, Gliwice, Poland) was dried over CaH_2_ and distilled under a dry argon atmosphere. Human dermal fibroblasts were derived from the Tissue Bank at the Dr. Stanislaw Sakiel Center for Burns Treatment in Siemianowice Slaskie. DMEM growth medium (Sterile A, Corning, NY, USA) was used with 10% FBS (Mediatech, Corning, NY, USA) and antibiotic/antimycotic (Gentamicin/Amphotercin B, Gibco, Fisher Scientific, Göteborg, Sweden). (3-(4,5-Dimethylthiazol-2-yl)-2,5-diphenyltetrazolium bromide) (MTT) (CyQUANT^TM^ MTT Cell Viability Assay Kit, Invitrogen by Thermo Fisher Scientific, Göteborg, Sweden), PBS (Sterile Filtered, Biowest, Kansas City, MO, USA) and DMSO (>99.9%, WAK–Chemie Medical GmbH, Steinbach, Germany) were used as received.

### 3.2. Synthesis of Copolymers

The molar ratio of the initial monomers concentration to the initiator concentration was 100:1. The composition of the comonomers EtMetOx:iPrOx was 10:90 and 50:50. Polymerizations were carried out at 75 °C in acetonitrile to the full conversion of the monomers (checked by gas chromatography). Then, water was added, the mixture was stirred for 10 min at room temperature, the excess acetonitrile was evaporated and the obtained polymers were dried by lyophilization. The copolymers were denoted as P(EtMetOx_10_-iPrOx_90_) and P(EtMetOx_50_-iPrOx_50_). The first copolymer was used for analyses, whilst the latter was used for cytotoxicity studies. Additionally, polymerization of EtOx and iPrOx was carried out with the same procedure. The composition of EtOx:iPrOx was set to 10:90. The obtained copolymer was denoted as P(EtOx_14_-iPrOx_86_) and its properties were compared to those of P(EtMetOx_10_-iPrOx_90_).

### 3.3. Measurements

The molar mass and dispersity (*Ð*) of the copolymers were determined using a GPC-MALLS system with a multiangle laser light scattering detector (DAWN EOS, Wyatt Technologies, Santa Barbara, CA, USA, λ = 658 nm) and a refractive index detector (Δn-1000 RI WGE DR Bures, Dallgow, Germany, λ = 620 nm). Measurements were carried out in DMF (with 5 mmol/L LiBr; flow rate of 1 mL/min) using PSS 100 Å, 1000 Å and 3000 Å GRAM columns.

The composition of the copolymers was analyzed by ^1^H NMR. The spectra were recorded in CDCl_3_ using a Bruker Ultrashield spectrometer (Bruker, Billerica, MA, USA) operating at 600 MHz.

DSC measurements were carried out using a TA-DSC Q2000 apparatus (TA Instruments, New Castle, DE, USA) under a nitrogen atmosphere with a flow rate of 50 mL/min. The measurements were taken in the range from 0 to 200 °C. The heating rate for the standard measurement was 10 °C/min and was equal to 2.5 °C/min for the so-called “slow-heating” measurement. To determine the glass transition temperature (T_g_) of the copolymer, after the first run, the sample was quenched with liquid nitrogen and a second heating run was performed again from 0 to 200 °C. The enthalpy of melting or crystallization (ΔH) was calculated as the area under the peak, limited by the baseline. The data were collected and then analyzed using Universal Analysis 2000 with Universal V4.5a software (TA Instruments, New Castle, DE, USA).

Turbidimetric measurements of the aqueous solutions of copolymers were performed using a Specord 200 plus UV-Vis spectrophotometer (Analytik Jena, Jena, Germany) equipped with a programmable thermocontroller. The transmittance of the polymer solutions was monitored at a wavelength λ = 550 nm as a function of the temperature (heating and then cooling cycle) with constant stirring of the solution. Transmittance values were recorded every 1 °C after 60 s of temperature stabilization. The concentrations of the polymer solutions ranged from 1 to 10 g/L. The phase transition temperature (T_CP_) was defined as the temperature at which the transmittance of the copolymer solutions reached 50% of its initial value.

The sample crystallinity was measured with a wide-angle X-ray diffractometer (WAXS) TUR-M62 (VEB TuR, Dresden, Germany) equipped with an HZG-3 goniometer (VEB TuR, Dresden, Germany) using Cu Kα radiation. Calculations of the intensities and positions of peaks were carried out using WAXSFIT software (WAXSFIT, Bielsko Biała, Poland).

### 3.4. Cytotoxicity Assay

The MTT cytotoxicity assay was used to estimate the cytotoxic properties of P(EtMetOx_50_-iPrOx_50_) in the treatment of human fibroblasts. Cells were seeded in a 96-well tissue culture microplate at a concentration of 3300 cells per well and incubated in 100 µL of growth medium at 37 °C and c(CO_2_) = 5% for 24 h. P(EtMetOx_50_-iPrOx_50_) was dissolved in the DMEM growth medium. Different concentrations of the polymer solution (0.001, 0.01, 0.1, 1 and 10 mg/mL) were added to the cells and the culture was carried out for 4, 8, 24, 48 and 72 h. A 12 mM MTT (3-(4,5-dimethylthiazol-2-yl)-2,5-diphenyltetrazolium bromide) stock solution was prepared in PBS. To detect formazan, a product of the redox reaction of viable cells, a rapid protocol using DMSO as a solubilizing agent was used. After a given culture time, the standard 100 µL of DMEM was replaced with DMEM without phenol red, as recommended by the MTT producer, because the presence of phenol red can affect the MTT assay results. Then, 10 μL of the 12 mM MTT stock solution was added to the well containing cells seeded in an appropriate concentration of the polymer and incubated at 37 °C for 4 h, followed by the removal of 85 µL of the medium. Fifty microliters of DMSO were added and pipetted up and down thoroughly for mixing. The microplate was incubated at 37 °C for 10 min. Then, the absorbance of the solution was measured at 540 nm using a Multiskan Sky Microplate Spectrophotometer (Thermo Scientific, Göteborg, Sweden). The number of cells was determined based on the calibration curve. The results are shown as a percentage of the control (cells seeded in DMEM without polymer).

## 4. Conclusions

Herein, we have shown that by the copolymerization of iPrOx with EtMetOx, it is possible to significantly suppress the ability of the copolymer to crystallize when compared to PiPrOx, whilst maintaining the LCST and T_g_ similar to those of PiPrOx. The appropriate choices of R_1_ (ethyl) and R_2_ (methyl) substituents of the comonomer used for copolymerization with iPrOx were crucial for controlling the properties of the final copolymer.

By the introduction of the R_1_ ethyl substituent of the comonomer and the control of amount of 2,4-disubstituted-2-oxazoline, the thermosensitivity could be controlled at the desired level. P(EtMetOx_10_-iPrOx_90_) exhibited a thermoresponsive behavior in water comparable to that of PiPrOx (no hysteresis of transition, LCST = 38 °C, a slight salting-out effect induced by the addition of NaCl).

The presence of the methyl substituent R_2_ randomly distributed within the main chain of the copolymer disrupted the chain regularity and led to a reduced ability of the copolymer to crystallize. Unlike PiPrOx, when long-term heating conditions in water were applied (12 h at 50 °C), no crystallization of the copolymer was observed, as revealed by turbidimetry. Additionally, P(EtMetOx_10_-iPrOx_90_) in the solid-state exhibited a definitely reduced ability to crystallize when compared to PiPrOx, whilst maintaining a T_g_ similar to that of PiPrOx, as confirmed by differential scanning calorimetry and X-ray diffractometry.

The copolymer was nontoxic to fibroblasts over a wide range of concentrations, which was confirmed by the calorimetric MTT assay.

On the basis of the obtained results, including the limited ability to crystallize, LCST close to the physiological temperature and T_g_ similar to that of PiPrOx, together with its nontoxicity to fibroblasts, we can conclude that P(EtMetOx_10_-iPrOx_90_) seems to have interesting prospects for many bioapplications, e.g., in tissue engineering or controlled drug delivery, and can clearly be an alternative to the “gold standard” thermoresponsive polymers.

## Data Availability

Data available on request.

## Supplementary Materials

The following are available online at https://www.mdpi.com/1422-0067/22/4/2221/s1.
