# Supplementary material for: Alternative to Poly(2-isopropyl-2-oxazoline) with a Reduced Ability to Crystallize and Physiological LCST
Source: Int J Mol Sci. 2021 Feb 23;22(4):2221. doi: 10.3390/ijms22042221 (PMC7926427; doi:10.3390/ijms22042221)
Supplement: Supplementary file 1 [file ijms-22-02221-s001.pdf]

*Supplementary Materials*

# Alternative to Poly(2-Isopropyl-2-Oxazoline) with a Reduced Ability to Crystallize and Physiological LCST

**Wojciech Wałach, Agnieszka Klama-Baryła, Anna Sitkowska, Agnieszka Kowalczuk and Natalia Oleszko-Torbus**

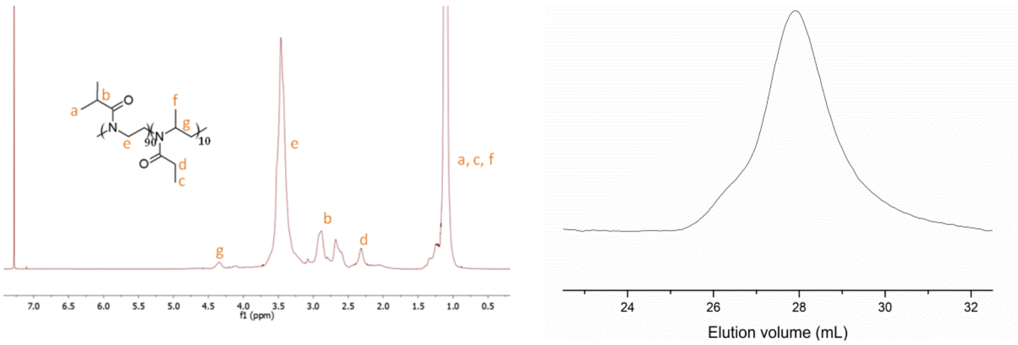


**Figure S1.** (**a**) ¹H NMR spectrum of P(EtMetOx$_{10}$-iPrOx$_{90}$) ($CDCl_3$); (**b**) SEC trace of P(EtMetOx$_{10}$-iPrOx$_{90}$) (DMF with 5 mmol LiBr as eluent, 1 mL/min, RI signal).

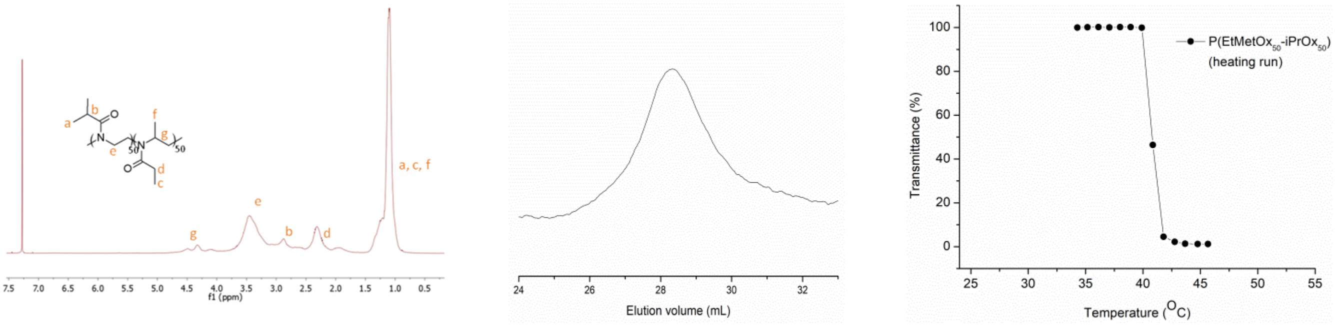


**Figure S2.** Characterization of P(EtMetOx$_{50}$-iPrOx$_{50}$): (**a**) ¹H NMR spectrum ($CDCl_3$); (**b**) SEC trace (DMF with 5 mmol LiBr as eluent, 1 mL/min, RI signal); (**c**) Transmittance-temperature dependence (aqueous solution, c = 5 g $L^{-1}$).

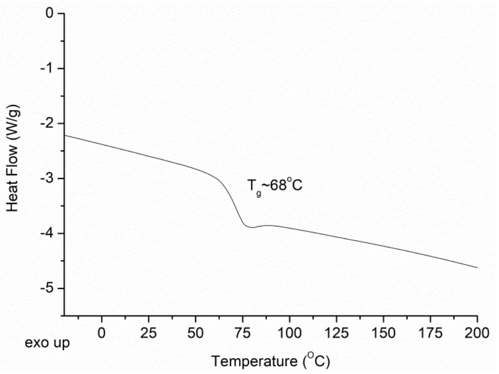


**Figure S3.** DSC trace of P(EtMetOx$_{10}$-iPrOx$_{90}$) after the first DSC run and quenching with liquid nitrogen.

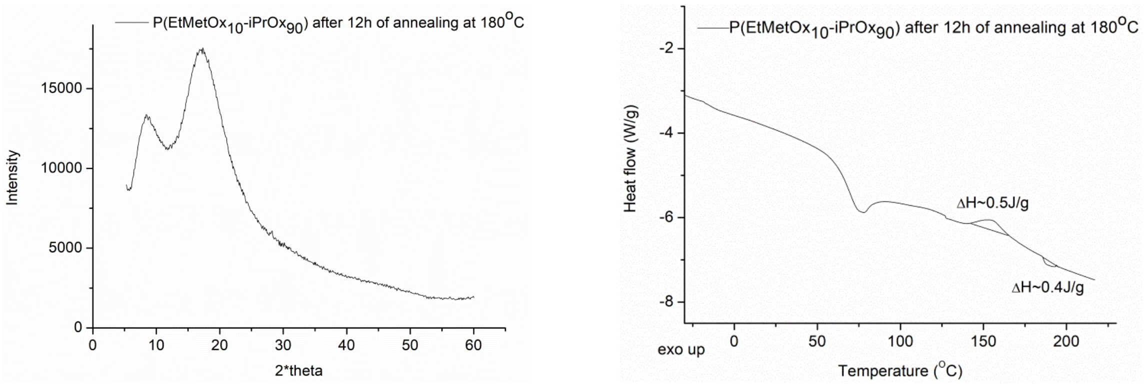


(a) (b)

**Figure S4.** P(EtMetOx10-iPrOx90) after annealing at 180 °C for 12 h and then cooling to room temperature at a rate of 10 °C/min: (**a**) X-ray diffraction curve; (**b**) DSC trace.

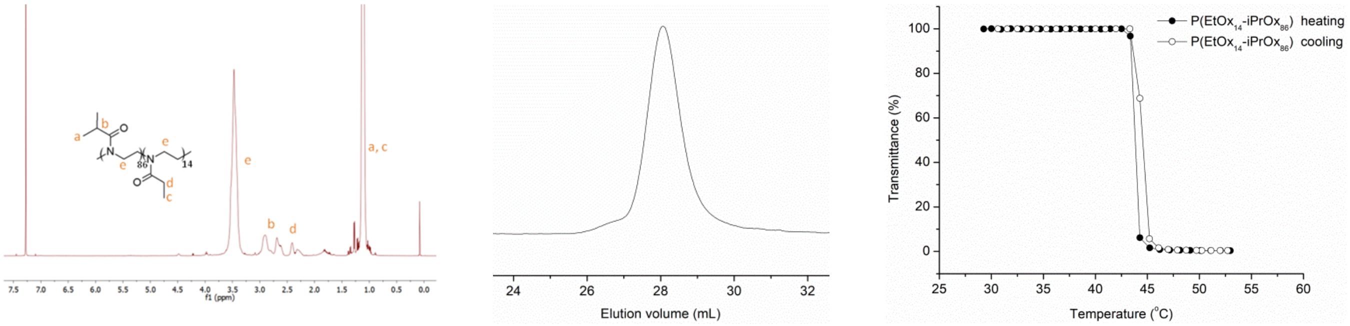


(a) (b) (c)

**Figure S5.** Characterization of P(EtOx14-iPrOx86): (**a**) $^1$H NMR spectrum ($CDCl_3$); (**b**) SEC trace (DMF with 5 mmol LiBr as eluent, 1 mL/min, RI signal); (**c**) Transmittance-temperature dependence (aqueous solution, c = 5 g $L^{-1}$).

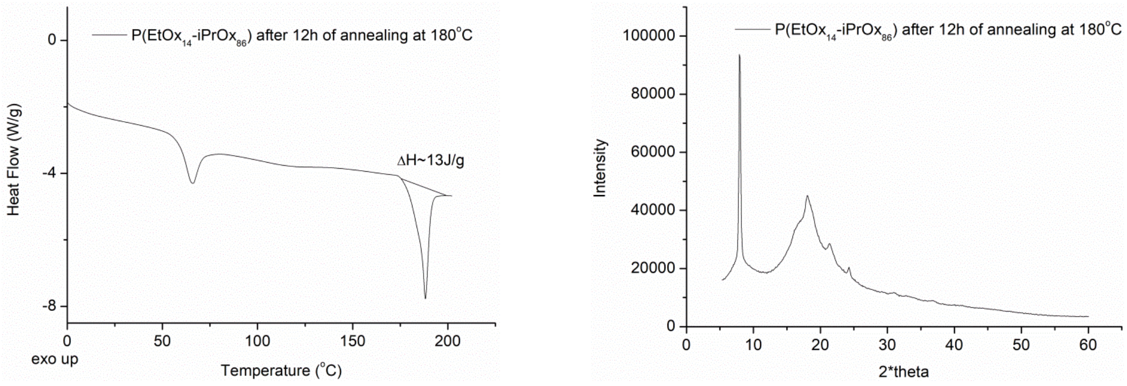


(a) (b)

**Figure S6.** P(EtOx14-iPrOx86) after annealing at 180 °C for 12 h and then cooling to room temperature at a rate of 10 °C/min: (**a**) DSC trace; (**b**) X-ray diffraction curve.
